# An international guideline training and certification programme

**DOI:** 10.2471/BLT.24.291587

**Published:** 2025-02-13

**Authors:** Holger J Schünemann, Patrick Okwen, Elie Akl, Paul Chrisp, Philipp Dahm, Yngve Falck-Ytter, Ivan D Florez, Markus Follmann, Elaine Harrow, Mouna Jameleddine, Miloslav Klugar, Miranda Langendam, Gillian Leng, Grigorios Leontiadis, Tamara Lotfi, Bogne Penka Marthe, Joerg Meerpohl, Zachary Munn, Ignacio Neumann, Thomas Piggott, Amir Qaseem, Nancy Santesso, Shahnaz Sultan, Wojtek Wiercioch, Jun Xia, Robby Nieuwlaat

**Affiliations:** a WHO Collaborating Centre for Evidence-Based Decision-Making in Health, Clinical Epidemiology and Research Centre, Humanitas University and IRCCS Humanitas Research Hospital, Via Rita Levi Montalcini 4, 20090 Pieve Emanuele, Milan, Italy.; b Effective Basic Services Africa, Bamenda, Cameroon.; c Department of Internal Medicine, American University of Beirut, Beirut, Lebanon.; d National Institute for Health and Care Excellence, Manchester, England.; e Department of Urology, University of Minnesota, Minneapolis, United States of America (USA).; f Case Western Reserve University, Cleveland, USA.; g Department of Pediatrics, Universidad de Antioquia, Medellin, Colombia.; h German Guideline Program in Oncology, German Cancer Society, Berlin, Germany.; i Guidelines International Network, Pitlochry, Scotland.; j Health Technology Assessment Department, National Authority for Assessment and Accreditation in Healthcare, Tunis, Tunisia.; k Institute of Health Information and Statistics of the Czech Republic, Prague, Czechia.; l Department of Epidemiology and Data Science, Amsterdam University Medical Center, Amsterdam, Kingdom of the Netherlands.; m Royal Society of Medicine, London, England.; n Department of Medicine, McMaster University, Hamilton, Canada.; o Department of Health Research Methods, Evidence and Impact, McMaster University, Hamilton, Canada.; p Faculty of Health Sciences, University of Cape Town, Rondebosch, South Africa.; q Institute for Evidence in Medicine, Faculty of Medicine, University of Freiburg, Freiburg, Germany.; r Health Evidence Synthesis, Recommendations and Impact, University of Adelaide, Adelaide, Australia.; s School of Medicine, Universidad San Sebastian, Santiago de Chile, Chile.; t American College of Physicians, Philadelphia, USA.; u Department of Medicine Division of Gastroenterology, Hepatology and Nutrition, University of Minnesota, Minneapolis, USA.; v Ningbo Nottingham GRADE Centre, University of Nottingham Ningbo China, Ningbo, China.

Trustworthy clinical, public health and health policy guidelines are products that set norms and standards based on the best available evidence. A single guideline, such as on tuberculosis or the coronavirus disease 2019 (COVID-19), can affect millions of people worldwide. Health guidelines are produced by the World Health Organization (WHO) and many other entities including the European Commission, health ministries, national health programmes, government organizations, professional societies and commercial organizations. To improve health outcomes and populations’ well-being, guidelines should optimize health workers’ actions, inform the public, create a basis for health policy and quality improvement and reduce unnecessary variation in care. However, although many evidence-based guidelines have been developed over the past 50 years, important gaps in quality of care remain. In addition to insufficient guideline implementation, at least two important global challenges create quality gaps in guideline development: lack of standardized guideline-related education leading to certification, and insufficient guideline creation capacity.

Consensus exists that guidelines should be developed by multidisciplinary guideline development groups that include individuals with specific skills or knowledge in a content area and people with lived conditions. Because creating guidelines is complex and requires expertise in the health and social sciences, credible guideline efforts are now routinely supported by guideline methodologists.^1^ A guideline methodologist has expertise in health research methods, in particular evidence syntheses and evidence-based decision-making, as well as group processes. Methodologists are included in guideline development groups at WHO.^2^ This inclusion, in part, was a result of a thorough evaluation of and recommendations to WHO about using evidence and developing standardized processes for guidelines.^1^ Additionally, many guideline developers have oversight committees to ensure guideline quality.^2^ Typically, the products of a guideline development group (that is, the recommendations) require contextualization to adequately consider baseline risks, values, equity, resources, acceptability and feasibility of interventions – particularly for guidelines developed for a global audience.^3^^,^^4^ However, a lack of appropriately trained guideline methodologists and guideline development group members, and uncertainty about the exact qualifications they should have,^3^^,^^4^ leads to a general lack of professionalism and excellence in the guideline enterprise.

## Training guideline leaders 

Currently, training for members of a guideline development group typically depends on those responsible for the guideline. As the field of guideline development is global and heterogenous, guideline developers have taken a variety of approaches (Box 1). At WHO, approaches include short informational sessions. WHO also provides training workshops and introductory webinars to some methodologists. However, WHO does not have the capacity to train all involved individuals, nor the mandate or capacity to develop guidelines on all topics. The agreed standards for guideline development^1^^,^^2^^,^^7^^,^^8^ are applied differently in training initiatives between jurisdictions, organizations and specialties, and, until recently, without certification to ensure necessary competency. For example, training programmes often do not carefully consider how to contextualize guidelines, or consider sensitive issues such as equity and acceptability of interventions and required social skills for consensus building.^9^ The low quality of COVID-19 guidelines produced across the globe are a stark demonstration of existing challenges.^10^

Box 1 **Selected examples of guideline training initiatives**

- WHO: WHO offers online learning material, such as for its tuberculosis guidelines through OpenWHO.
- Professional groups, societies and organizations: In a project to create 10 guidelines for the American Society of Hematology, a team of guideline methodologists prepared training material for future guidelines and led the guideline development group and the overall process^5^ including the adaptation of the guidelines in South America.^6^ The European Respiratory Society has a clinical practice guideline method network to support its guideline programme. The Guideline International Network and professional societies regularly offer training courses at annual meetings. The GRADE Working Group with its over 30 global networks and centres offer variable training opportunities, including courses and long-term methods support, across the globe.
- National entities: Germany offers short courses and workshops as part of the national guideline oversight by the Association of Scientific Medical Societies focused on methods.
- Academia: Graduate courses in guideline methods, such as those offered by McMaster University.

GRADE: Grading of Recommendations, Assessment, Development and Evaluations; WHO: World Health Organization.

## Guideline certification for quality 

One way to ensure the guideline sector follows international standards^1^^,^^2^^,^^7^^,^^8^ is to provide certified training to involved individuals and then certify the trainees and products. Recently, key organizations started to address the issue of professionalism and certification in the guideline enterprise. The National Institute for Health and Care Excellence in the United Kingdom of Great Britain and Northern Ireland; the European Commission in its initiatives on cancer; the Public Health Agency of Canada; the German Association of Scientific Medical Societies; the European Academy of Allergy and Clinical Immunology; the Endocrine Society; and the American College of Physicians embraced a new level of guideline quality assurance. These organizations started to train methodologists and guideline development group members in the international guideline training and certification programme INGUIDE. This unique programme, delivered under the auspices of the Guidelines International Network, addresses the critical need for standardization, quality improvement and capacity-building in health guideline development and effective implementation.^11^ The training programme provides evidence-based training and can provide certification to all individuals involved in the guideline enterprise, including those with lived experiences. The programme also promotes the systematic development of guidelines, based on the best available evidence and adherence to international quality standards, including training in the Grading of Recommendations, Assessment, Development and Evaluations (GRADE) method.^11^^,^^12^ Over 2000 guideline development group or panel members, methodologists and instructors from different backgrounds have been trained, including in experiential, competency-based components as described in detail elsewhere.^11^ The programme, working alongside WHO and other organizations, is committed to adapting its content and methods to diverse health-care contexts, particularly in low- and middle-income countries, to strengthen capacity.^13^ Therefore, the programme is facilitating the translation of relevant material and offering specialized modules, such as GRADE adolopment, to strengthen capacity in adapting guidelines to a country level.^3^^,^^4^ This capacity strengthening will help support the production of guidelines at WHO and other structures that seek to develop guidelines with or for low- and middle-income countries. The programme also helps connecting with other actors for evidence-based decision-making.^14^ However, this training programme is only part of the solution. Those who are responsible for the institutionalization, creation and use of guidelines, at all levels, must commit to quality and capacity. This commitment requires awareness of the challenges related to guidelines in and across health systems in a culture and environment of quality improvement that is incentivized. For example, in jurisdictions where guideline development comes through a national mandate, policy-makers have to identify the problem and assess relevant needs before making investments to take appropriate action for certification, capacity strengthening and resource allocation for guidelines.^9^ This capacity strengthening should include those who have an interest in guidelines, such as health workers, professional societies, government organizations and the public, in inclusive ways.^3^

## Challenges in certification

Our suggested solutions are not without challenges. No systematic information about the programme’s impact is available yet. Assessing the impact of training on health outcomes is challenging because of the many confounding factors. Challenges also exist with conducting experiments measuring health outcomes. Thus, impact evaluation could focus on knowledge, attitude, satisfaction and skills, both qualitatively and quantitatively. Concerns have also been raised about the cost involved in certification. Any training programme requires resources, even if training is offered for free. Certification requires ongoing resources and funding needed to maintain a database of certified individuals and to fulfil the International Organization for Standardization’s (ISO) 9001 certification requirements for the quality control of the programme. INGUIDE is dedicated to obtaining unrestricted financial support from funders to provide equitable access to its benefits and implement guideline certification broadly. For example, Humanitas University in Italy sponsors learners from low- and middle-income countries selected with WHO to strengthen relevant capacity. Training must be tailored to the needs and context of the target group, empowering learners to apply their skills in their environment. A possible solution might be adopting a flexible approach to training while ensuring certification in core competencies. Addressing the concern of whether guidelines are needed requires dialogue with policy-makers to ensure they are aware of the role of guidelines in health decision-making.^14^ Policy-makers should also consider that guidelines improve outcomes and are wanted by practitioners to guide care, while empowering people to make informed decisions.

## Next steps 

Professionalizing guideline training and its certification is crucial for enhancing the trustworthiness, quality, effectiveness and global reach of health guidelines. Given the breadth of the field of guideline development and science, WHO cannot accomplish this goal alone. INGUIDE seeks to professionalize the overall guideline enterprise, similar to other health professions. The programme has already organized mentorship for methodologists at WHO, such as those at the Global Tuberculosis Programme, and trained members of guideline development groups at WHO – but not consistently reaching all guideline methodologists. Further collaboration with WHO and other entities could enhance the impact of certification. Professionalizing guideline training and certification in low- and middle-income countries will enable trustworthy guidelines to respond to local needs and address the guideline quality gap observed in some low- and middle-income countries.^15^

We call for guideline developers to support and adopt certification with a focus on quality to strengthen capacity. Training should be rigorous and give trainees enough capacity and freedom to include contextual needs, be independent from external influences and develop quality guidelines that respond to country priorities. In addition to enhancing the trustworthiness of guidelines, education in guideline development and implementation creates knowledge and capacity in “everything evidence” in health research. With “everything evidence”, we refer to delving into the comprehensive scope of evidence-based practices, methods, types of evidence and the principles guiding the integration of research findings into health decision-making. This knowledge and capacity goes beyond health workers and includes people affected by the conditions which the guidelines are designed to address. Those supporting capacity strengthening in related sectors should consider that elevating health guideline development standards worldwide is a priority for reaching the sustainable development goals. INGUIDE could be expanded to other norms and standard-setting products such as health technology assessment.
